# Influenza-associated in-hospital mortality during the 2017/2018 influenza season: a retrospective multicentre cohort study in central Germany

**DOI:** 10.1007/s15010-020-01529-x

**Published:** 2020-09-27

**Authors:** Steve Rößler, Juliane Ankert, Michael Baier, Mathias W. Pletz, Stefan Hagel

**Affiliations:** 1 Institute for Infectious Diseases and Infection Control, Jena University Hospital, Am Klinikum 1, 07747 Jena, Germany; 2 Institute of Medical Microbiology, Jena University Hospital, Jena, Germany

**Keywords:** Influenza, In-hospital mortality, Season 2017/2018, Intensive care

## Abstract

The aim of this retrospective cohort study at eight hospitals in Germany was to specify influenza-associated in-hospital mortality during the 2017/2018 flu season, which was the strongest in Germany in the past 30 years. A total of 1560 patients were included in the study. Overall, in-hospital mortality was 6.7% (*n* = 103), in patients treated in the intensive care unit (*n* = 161) mortality was 22.4%. The proportion of deceased patients per hospital was between 0% and 7.0%. Influenza was the immediate cause of death in 82.8% (*n* = 82) of the decedents.

## Purpose

It is estimated that 5–20% of the population contracts influenza in an average flu season [1]. Among the 30 most relevant infectious diseases in Europe, influenza, with 5.9 infections per 100,000 inhabitants annually, has the highest incidence and the highest mortality (5.89 deaths per 100,000 inhabitants per year) [2]. Approximately one-third of the total burden of all infectious diseases, measured in disability-adjusted life years, is attributable to influenza [2]. However, it has been recognized for many years that influenza is underreported on death certificates. It is, therefore, common practice to estimate mortality attributed to influenza with statistical methods [3]. This so-called excess mortality is obtained by subtracting the expected mortality (i.e. background mortality) from the observed mortality during an influenza season. If an increase in mortality is observed which is significantly higher than background mortality, this is attributed to influenza. For example, according to the Robert Koch Institute (RKI), an estimated 25,100 people in Germany died due to influenza during the 2017/2018 flu season, but only 1674 influenza-associated deaths were officially reported [3]. As in-hospital mortality is not collected as part of RKI influenza surveillance, no data are available on this. However, accurate data on the burden of disease are important for making comparisons with other diseases, e.g., Coronavirus disease 2019 (COVID-19) [4]. Therefore, the primary aim of this study was to specify influenza-associated in-hospital mortality during the 2017/2018 influenza season, the strongest influenza season in the past 30 years. In addition, the need for intensive care support was assessed, an important number to describe severity of disease.

## Methods

This retrospective cohort study was performed at eight hospitals with 4976 beds (median 366 (range 80–1735) beds) in central Germany (Table 1). Six hospitals were small regional hospitals providing basic and standard care, and the two largest hospitals were hospitals providing maximum care. All hospitalized patients with a discharge diagnosis of influenza infection (J.10.-) between December 2017 and April 2018 were included in the analysis. In addition to demographics, length of hospital stay, intensive care unit (ICU) stay and hospital discharge data were collected from all patients. In patients who died during their hospital stay, chart review was performed to document information about comorbidities, the cause of death and therapy. Immunosuppression was defined as the presence of congenital or acquired immunosuppression (e.g., chronic therapy with corticosteroids > 10 mg/day, radio/chemotherapy, transplantation, HIV/AIDS). To summarize characteristics of deceased patients, we provide absolute and relative frequencies and the median complemented by the first and third quartiles (Q1, Q3). We applied the *χ*^2^ test or, if indicated, Fisher’s exact test and the Mann–Whitney *U* test to assess differences between deceased patients with ICU care and without ICU care. Ethical approval for the study was provided by the Ethics Committee of the Jena University Hospital, with a waiver for informed consent from the patients (5532-05/18).

**Table 1:** Hospital size, number of patients with influenza per hospital and influenza-associated mortality

Hospital	Beds (*n*)	Patients with influenza, overall, *n* (% of total number)	Deceased patients with influenza, *n* (% of hospitalized patients)
1	313	18 (1.2)	0 (0)
2	420	7 (0.5)	0 (0)
3	212	116 (7.5)	4 (3.4)
4	285	48 (3.1)	4 (8.3)
5	80	16 (1.0)	1 (6.3)
6	535	256 (16.6)	14 (5.5)
7	1396	402 (26.1)	29 (7.2)
8	1735	676 (43.9)	51 (7.5)
Overall	4976	1539 (100)	103 (6.7)

## Results

A total of 1560 patients were diagnosed with influenza infection during the study period. In 21 patients, data were incomplete; therefore, 1539 patients were included in the subsequent analysis. Table 1 shows an overview of the number of influenza cases per hospital. The median age of the patients was 72 (range 0–102) years, and 769 (50%) patients were male. The average length of hospital stay was 11.4 ± 13.4 days (median 7 days, range 0–130 days). Overall, 103 [6.7%, 95% confidence interval (CI) 5.5–8.1%] out of 1539 patients died during their hospital stay. The proportion of deceased patients per hospital was between 0% and 7.0% (Table 1). In 82.8% (*n* = 82) of the cases, influenza infection was considered to be the direct cause of death; in 15.2% (*n* = 15) of the cases, it was an indirect cause of death; and in 2% (*n* = 2) of the cases, it did not affect patient death.

### Intensive care

In total, 161 patients (10.3%) were treated in an ICU during their hospital stay. The median age of these patients was 72 (range 32–96) years, and 60% (*n* = 96) were male. Detailed information on the course of the disease was obtained from 111 patients receiving intensive medical care. In 44.1% (*n* = 49) of the patients, influenza infection was the immediate and primary reason for admission to the ICU; in 13.5% (*n* = 15) of patients, infection was acquired in the ICU. Influenza infection was present in 20.7% (*n* = 23) of patients, but it had no influence on admission to the ICU. In another 21.6% (*n* = 24) of patients, influenza infection worsened the underlying illness, leading to ICU admission. Extracorporeal membrane oxygenation was successfully performed in one patient. A total of 36 patients (22.4%, 95% CI 16.1–29.6) died during their stay in the ICU. Influenza infection was the immediate cause of death in 29 patients (80.6%) and an indirect cause in 7 (19.4%) patients. Influenza subtyping was performed in 97 patients in the ICU. Sixty-seven patients (69.1%) had influenza B infection, and 30 patients (30.9%) had influenza A infection (*p* < 0.001) (Table 2).

**Table 2:** Clinical and demographic characteristics of all deceased patients with influenza (column 1) and deceased patients with (column 2) and without (column 3) the need for intensive care during hospitalization

	Deceased patients, total (*n* = 103)^c^	Deceased patients, with ICU care (*n* = 36)	Deceased patients, without ICU care (*n* = 67)^c^	*p*^b^
Age (years), median (Q1, Q3)	78 (70, 86)	73 (67, 77)	82 (75, 90)	< 0.001
Sex				
Male, *n* (%)	57 (55.3)	23 (63.9)	34 (50.7)	0.145
Female, *n* (%)	46 (44.7)	13 (36.1)	33 (49.3)	0.003
Chronic pulmonary disease, *n* (%)				
COPD	22 (22.2%)	11 (30.6)	11 (17.5)	1000
other	9 (9.1)	4 (11.1)	5 (7.9)	0.739
Chronic heart failure, *n* (%)	42 (42.4)	14 (38.9)	28 (44.4)	0.031
Obesity, *n* (%)	5 (5.1)	2 (5.6)	3 (4.8)	0.655
Neurological diseases, *n* (%)	34 (34.3)	8 (22.2)	26 (41.3)	0.002
Chronic haemodialysis, *n* (%)	7 (7.1)	4 (11.1)	3 (4.8)	0.705
Malignancy, *n* (%)				
With chemotherapy	8 (8.1)	4 (11.1)	4 (6.3)	1.000
Without chemotherapy	13 (13.1)	4 (11.1)	9 (14.3)	0.166
Immunsuppression^a^, *n* (%)	7 (7.1)	3 (8.3)	4 (6.3)	0.705
Influenza-type, *n* (%)				
A	15 (15.2)	8 (22.2)	7 (11.1)	0.796
B	58 (58.6)	17 (47.2)	41 (65.1)	0.002
Unknown	26 (26.3)	11 (30.6)	15 (23.8)	
Length of hospital stay (days), median (Q1, Q3)	9.0 (4.0, 17.0)	17.5 (11.0, 34.3)	6.0 (3.0, 11.0)	< 0.001
Therapy				
Oseltamivir, *n* (%)	46 (46.5)	24 (66.7)	22 (34.9)	0.768
Intensive care, *n* (%)	36 (35.0)	36 (100)	0 (0.0)	–
Mechanical ventilation, *n* (%)	36 (36.4)	36 (100)	0 (0.0)	–
Acute renal replacement therapy, *n* (%)	14 (14.1)	12 (33.3)	2 (3.2)	0.008
Therapy limitation, *n* (%)	28 (28.3)	3 (8.3)	25 (39.7)	< 0.001

^a^Without malignancy

^b^Difference between patients “with” and “without” intensive care

^c^Except for age, sex, duration of hospital stay and intensive care, data of 4 patients are missing

## Discussion

During the 2017/18 influenza season, approximately 334,000 laboratory-confirmed influenza cases were reported to the RKI. Approximately 60,000 (17%) patients were reported to be hospitalized. In the present study involving eight hospitals in central Germany, influenza-related in-hospital mortality in the examined patient population was 6.7%, and every tenth patient was cared for in an ICU during their hospital stay. The in-hospital mortality in this group of patients was significantly higher (22.4%) than that in those who did not require admission to an ICU. Our observed in-hospital mortality rate of 6.7% was comparable to the in-hospital mortality rate of 5.8% reported by another German university hospital [5] but lower than the in-hospital mortality (8.3%) rate reported by a tertiary hospital in Austria [6]. Interestingly, the in-hospital mortality observed in this season was significantly lower compared to the in-hospital mortality in the 2014/2015 season (7.0% vs. 10.4%) in study hospital No. 7 [7].

Applying our observed in-hospital mortality of 6.7% to the 60,000 hospitalized patients with a laboratory-confirmed influenza infection reported to the RKI would correspond to approximately 4000 deceased hospitalized patients during the 2017/2018 flu season in Germany. This in contrast to the 1674 influenza-associated deaths officially reported to the RKI, for both, hospitalized and non-hospitalized patients, clearly underlying the notion that influenza-associated deaths are underreported. However, our observed in-hospital mortality does not allow to draw conclusions about the overall influenza-associated mortality and burden of disease. For one thing, the mortality in non-hospitalized patients with influenza most probably differs from hospitalized patients with influenza. On the other hand, the overall number of patients infected with influenza each season is unknown. Not every person who truly has influenza will seek medical care, will be tested for influenza, have a positive test, and, therefore, be reported through influenza surveillance [8]. Routinely available influenza diagnostic tests also vary in sensitivity. Thus, data collected through influenza surveillance and case finding represent only a fraction of persons infected with influenza. For example, a recently published study calculated that in the US, only between 0.08–0.61% and 0.07–0.33% of symptomatic influenza illnesses were laboratory-confirmed in the 2011–2012 and 2012–2013 seasons, respectively [8].

In 1129 (67.4%) influenza-associated deaths reported to the RKI, it was stated that the patient died from influenza disease and its consequences [3]. This proportion was lower than that in our study, in which influenza infection was considered to be the immediate cause of death in 82.8% of the deceased patients. This discrepancy could be because, unlike in the current study in which a doctor made a decision regarding whether death was associated with influenza infection based on a medical chart review, the health authorities made the decision solely on the basis of the information available to them. These decisions considered assessments by the supervising doctor or information on the death certificate. However, these types of assessments are problematic because, in contrast to other diseases, influenza is often not recorded on the death certificate as the cause of death, even if influenza had been confirmed by laboratory testing in the course of the disease.

### Limitations

There are several limitations in the current study. Patients with influenza infection were identified retrospectively on the basis of the diagnosis code at discharge. Since it is possible that not every microbiologically confirmed influenza infection was coded at discharge and that microbiological diagnostics were not always performed to ensure the clinically suspected diagnosis, the true number of hospitalized influenza patients could be correspondingly higher than that reported here. In addition, as the assessment of whether the death of a patient was related to influenza infection was made by a study physician, the results may be subject to bias. Nevertheless, the results of the present study underline the high burden of disease in hospitalized patients with influenza and allow a comparisons with other diseases, e.g., Coronavirus disease 2019 (COVID-19).
